# Rethinking Human Resources for Health Planning in Labour Markets Disrupted by Conflict-Affected and Fragile Settings: Comment on "Human Resources for Health in Conflict Affected Settings: A Scoping Review of Primary Peer Reviewed Publications 2016–2022"

**DOI:** 10.34172/ijhpm.9534

**Published:** 2026-02-08

**Authors:** Roomi Aziz

**Affiliations:** Centre for Global Health and Intersectional Equity Research, University of Essex, Colchester, UK.

**Keywords:** Human Resources for Health, Conflict-Affected Settings, Fragile Health Systems, Health Labour Market

## Abstract

In a world still grappling with exploring the underlying dynamics of challenges confronting human resources for health (HRH), how must the HRH research and planning ensue in conflict-affected settings (CAS)? Onvlee and colleagues undertake a scoping review to respond to this important question, using the World Health Organization’s (WHO’s) Health Labour Market (HLM) framework, to leverage upon available evidence. This commentary appraises the conceptual and methodological contributions of the review, while questioning the suitability of HLM to analyse HRH challenges in disrupted health systems. It argues that CAS-specific HRH planning exacts frameworks and approaches more attuned to political economy, contextual fragility, and structural inequalities, which shape healthcare workers’ vulnerabilities and responses in CAS. The commentary identifies five gap questions for future scholarship, calling for intersectionality-driven, politically informed and context-specific research approaches for HRH evidence, transcending supply and demand framing of HRH, to inform HRH policies in conflict-affected and fragile settings.

## Planning for Human Resources for Health in Conflict-Affected Settings: A Critical Research Priority

 There is a growing interest in studying human resources for health (HRH) crisis in specific situations, to identify and invest in very niche and bespoke policy solutions, especially in conflict-affected settings (CAS)^1^. In this context, the scoping review by Onvlee et al, “Human Resources for Health in Conflict Affected Settings: A Scoping Review of Primary Peer Reviewed Publications 2016–2022,” is a valuable and timely contribution. The review provides a very relevant synthesis of global evidence around HRH challenges and policy-making in CAS,^2^ using the Sousa’s Health Labour Market (HLM) framework.^3^ By foregrounding how healthcare workers’ (HCWs’) experiences are shaped by conflict dynamics, this review places HRH planning on the policy agenda for post-conflict health systems’ re-building and resilience. Today this review is even more relevant as globally conflicts and wars have completely obliterated local health landscape while also morphing the local burden of disease.

 This commentary appraises the conceptual and analytical strengths of the respective scoping review, while advancing a central argument: although the HLM framework offers a structured organising scaffold to present an analysis of the HRH system, its application in CAS remains analytically limited. This can only work when it is paired with approaches engaging governance fragmentation, political economy and other structural inequalities. This commentary builds on some of the challenges identified in the review, and draws on insights developed from other similar works to outline a focussed research agenda for advancing HRH scholarship and policy learnings in CAS and fragile settings.

## What Works in This Review?

 The review has made three notable contributions. First, the authors have introduced a conceptual innovation, by structuring their findings using an adapted version of Sousa’s HLM framework, for HRH planning in fragile conflict settings.^3^ Onvlee and colleagues’ review builds upon Bou-Karroum and colleagues’ systematic review, addressing multiple gaps in the same^4^. While the earlier systematic review was broader and more global in scope, it remained largely descriptive, focussing on bibliometric analysis and visual mapping.^4^ HRH planning in CAS settings is very different from that in stable contexts, especially as experienced differently by HCWs’ different positionalities and intersecting identities, including their gender, ethnicity, cadre etc. Recognising the needs of an unstable context, the authors incorporate a governance and context layer to the HLM framework to present their analysis.

 Second, through the context layer introduced in their adaptation of the HLM framework, the authors have identified how the changing positionalities of HCWs and their intersecting identities; due to their gender, ethnicity, race, profession, and class; shape their exposure to risks and the challenges they experience in CAS. This acknowledgment is also gathering traction in literature.^5^ This includes the heightened targeting of HCWs due to their professional status, ie, “weaponisation” of health, whereby HCWs themselves become objects of coercion, intimidation or attacks by conflict actors. The review also draws attention to gender-based violence and gendered vulnerabilities in conflicts and humanitarian crisis, echoing concerns highlighted in the World Health Organization (WHO) HRH reader.^1^

 Third, application of governance lens strengthens the review’s policy relevance. The authors explain how CAS dynamics multiply and compound the otherwise common HRH challenges in Low- and middle-income country context, extracting lessons for governments and development partners operating in CAS. Specifically, it underscores the need of context-aware HRH policy-making for mid- and long-term around HCWs’ safety and protection, not just their management or distribution. The authors highlight how the stopgap arrangements by international non-governmental organisations (INGOs) and non-governmental organisations** (**NGOs) to attract and deploy HRH may temporarily sustain services but cease to function post-disasters and conflicts. This temporariness hinders sustainable and long-serving policy solutions.

## Contribution of This Review – Segmenting Human Resources for Health Challenges Unpacked Along the Health Labour Market in Conflicts and Humanitarian Crisis

 In line with the HLM framework, the review has systematically identified how conflict disrupts HRH across education, labour market dynamics and governance. The review highlights how education and training pathways are compromised through damaged infrastructure, inadequate accreditation, faculty shortage, compromising workforce preparedness, and creating long-term imbalances.

 At the labour market, the review underscores recurrent patterns of HCWs’ outflow via internal displacement, internal movement to urban centres, international migration and health sector exit recruitment challenges, leading to reliance on community health workers, and rapid and inappropriate deployment relying on non-state actors like NGOs, donors working on the ground as parallel systems. These movements are not only driven by insecurity, but also due to irregularity in remuneration, poor working conditions, and the pull of better-paying humanitarian organisations. Managing performance of the HCWs who remain also emerged as a challenge, with disruption in regular supervision and professional development opportunities. The HCWs left behind often have to tackle more difficult cases while not being prepared for them, resulting in compromised quality of care. Even repatriating HCWs who left the country earlier, results in resentments within local HCWs due to salary differentials, limited opportunities for local HCWs, inability of repats to transfer skills locally and lack of training gaps.^6^ The authors also acknowledge limitations of the included papers focussing on HCWs left behind in CAS and not those who have left, which implies missing the perspective of otherwise critical HCWs.

 The authors also identified cross-cutting HRH governance challenges, such as gaps due to institutional fragmentation, weak coordination, donor engagement and over-reliance resulting in consistently weak state machinery and erosion of public trust. Within this context layer, HCWs become targets themselves, or collateral damage, making their physical and psychological safety a central concern for HRH planning in CAS.

## Adequacy of the Health Labour Market Framework for Analysing Human Resources for Health in Conflict-Affected Settings

 Onvlee et al recognise how reconstruction frameworks for post-conflict settings neglect conflict-specific issues, including monitoring, coordination, need of legal frameworks, all important from conflict point of view.^7^ The review closes with need of context-aware HRH policy-making around re-distributing, retaining, financially incentivising, and supporting HCWs, and protecting them. However, the review’s use of HLM framework raises an important analytical question: To what extent is the HLM framework adequate for understanding HRH dynamics in protracted conflict and fragility. The review acknowledges lack of broad-based policy-relevant evidence, absence of long-term view on HLM dynamics, limited focus on comprehensive coverage, causes and consequences of fragmentation and disarray due to conflicts. However, the HLM framework is implicitly rooted in a stable setting with functional institutions for education, accreditation, deployment, remuneration and regulation, with governance structures capable of steering HCWs policies. In CAS, these assumptions all stand violated. Health systems are operating in CAS, but with disrupted infrastructure, fragmented authorities, stopgap service delivery arrangements and highly unstable financing. HCWs’ mobility, retention and attrition are shaped by political control, macroeconomic situation, security and survival strategies more than conventional labour market dynamics.

 Most of the existing reviews have used supply-oriented frameworks like HLM or human resource management, which overlook power, politics or governance. Onvlee et al recognise absence of focus on human resource management governance and introduce conflict and governance layers to their application of HLM framework.^8^ However, these layers function as contextual modifiers, missing key dynamics like distorted feedback loops, non-market control, political and structural drivers of vulnerabilities and inequities. Viewing through these frameworks inadvertently results in identification of training as a dominant HRH intervention focus. This review applies HLM while explicitly acknowledges governance challenges and parallel systems on ground in CAS, but this application is better suited as a descriptive skeleton rather than an analytical lens for HRH under protracted fragility and CAS.

## Advancing Human Resources for Health Research and Policy-Making in Conflict-Affected Settings – Five Gap Questions

 This commentary identifies five questions pertinent to this review that remain unanswered or partially answered. Responding to these questions would be in the interest of scholarly dialogue and advancing HRH scholarship and CAS-specific health systems strengthening.

###  1. How Is Human Resources for Health Research Happening in Conflict-Affected Settings?

 The review recognises paucity of research in CAS but does not map the methodological approaches used in the included studies. The review also acknowledges the role played by humanitarian organisations and INGOs in conflict settings, but does not include grey and unpublished literature, especially NGO and INGO reports. Methodology mapping is a key area for future scholarship to focus on, examining how insecurity, displacement and ethical constraints shape research design and choices, as well as research producers.

###  2. How Has Human Resources for Health Scholarship in Conflict-Affected Settings Changed Since the 2020 Systematic Review?

 While addressing synthesis gaps in Bou-Karroum and colleagues’ review,^4^ this scoping review does not inform whether the scholarship has diversified vis-à-vis authorship, geographic coverage or thematic focus. This review also uses a much leaner search string for HRH, missing out on a lot of other relevant literature. Acknowledging that most of the evidence has had a narrow focus on a particular cadre or situation, more expansive search strategies are required to capture cadre- and context-specific evidence for policy-making.

###  3. Is HLM an Appropriate Analytical Framework for Use in Conflict-Affected Settings?

 The review recognises that planning in CAS is very different than in stable country states, with distinctly different starting points, disrupted systems and fragmented governance. Recovery opportunities come post-conflict, prioritising stability, protecting HRH and reconstructing the system.^9^ While the authors did introduce a conflict layer to the HLM framework, the layer has been treated as a modifier rather than a systemic disrupter. Bertone et al also talk about stages of policy-making in CAS, the political situation uncertainty, availability of resources, sense of radical change and highly varying timelines,^10^ all of which are not captured within the HLM framework in its current design. In protracted fragility, supply-based frameworks like HLM need to be supplemented with political economy lens and systemic drivers that explicitly address power coercion, institutional breakdown and non-linear recovery trajectories.^11^

###  4. How Could Equity Considerations Be Captured in the Literature?

 Although the authors noted gendered vulnerabilities, they did not explicitly use an intersectional lens to present complexed nuances along gender, race or cadre, and their intersection with poverty and household structure. This is particularly critical to understand the impact of intersecting axes of inequities due to structural discrimination in fragile and shock settings and plan HRH research and evidence design accordingly. New conceptual clarifications of HRH crisis also recognise structural discrimination is as its third dimension.^12,13^

###  5. What Is the Best Way to Find Conflict-Affected Settings-Relevant Human Resources for Health Policy and Programmatic Interventions

 The review concludes that evidence on HRH interventions is limited and fragmented, and exacts that HRH policies must be aligned to political and broader societal context, including conflict dynamics. However, if specific market policy-levers are the desired evidence output, future reviews would benefit from explicit search on policy-making in post-conflict settings, political analysis to unpack the fragmentated policy landscape in the country, as well as both centralised and decentralised health and HRH functions. Looking at politics, actors, instruments in conflict-settings will allow bringing forth a change in HRH policy-making by disrupting the existing order.^14^

## Conclusion: How to Go About Human Resources for Health Research in Conflict-Affected Settings From Here Onwards?

 As reiterated by the authors, this review has only surfaced evidence and policy gaps around HRH planning in CAS but it does put forth an important analytical synthesis around HRH research in CAS. It does this by foregrounding governance dynamics often ignored in earlier works, while identifying gaps and focus for future reviews. This commentary has raised five gap questions, together with the original review’s closing recommendations, consolidated into following actionable implications for future HRH scholarship and policy learnings specific to CAS settings.

- Evidence sources must be broadened and triangulated while maintaining analytical rigour;
- HLM-style frameworks must be paired with analysis of political economy, systems and governance;
- Intersectional equity considerations must be institutionalised in HRH data and research;
- For CAS research, types of conflicts must be thoughtfully defined and staged to identify specific and responsive policy solutions; and finally
- Future HRH in CAS scholarship must move beyond stock-and-flow models, towards frameworks that capture politics, local contexts, precarities, lived realities and inequities experienced by HCWs.

## Disclosure of artificial intelligence (AI) use

 Not applicable.

## Ethical issues

 Not applicable.

## Conflicts of interest

 Author declares that she has no conflicts of interest.
